# Light/dark phase-dependent spontaneous activity is maintained in dopamine-deficient mice

**DOI:** 10.1186/s13041-017-0329-4

**Published:** 2017-10-16

**Authors:** Masayo Fujita, Yoko Hagino, Taishi Takeda, Shinya Kasai, Miho Tanaka, Yukio Takamatsu, Kazuto Kobayashi, Kazutaka Ikeda

**Affiliations:** 1 Addictive Substance Project, Tokyo Metropolitan Institute of Medical Science, 2-1-6 Kamikitazawa, Setagaya-ku, Tokyo, 156-8506 Japan; 2 Center for Basic Technology Research, Tokyo Metropolitan Institute of Medical Science, 2-1-6 Kamikitazawa, Setagaya-ku, Tokyo, 156-8506 Japan; 3 Department of Molecular Genetics, Institute of Biomedical Sciences, Fukushima Medical University, 1 Hikarigaoka, Fukushima, 960-1295 Japan

**Keywords:** Dopamine, Dopamine-deficient mice, Light/dark phase, Spontaneous activity

## Abstract

Dopamine is important for motor control and involved in the regulation of circadian rhythm. We previously found that dopamine-deficient (DD) mice became hyperactive in a novel environment 72 h after the last injection of L-3,4-dihydroxyphenylalanine (L-DOPA) when dopamine was almost completely depleted. DD mice did not initially exhibit hyperactivity in their home cages, but the animals exhibited hyperactivity several hours after the last L-DOPA injection. The regulation of motor activity in a novel environment and in home cages may be different. A previous study reported that DD mice became active again approximately 24 h after the last L-DOPA injection. One speculation was that light/dark phase-dependent spontaneous activity might be maintained despite dopamine deficiency. The present study investigated whether spontaneous home cage activity is maintained in DD mice 24–43 h and 72–91 h after the last L-DOPA injection. Spontaneous activity was almost completely suppressed during the light phase of the light/dark cycle in DD mice 24 and 72 h after the last L-DOPA injection. After the dark phase began, DD mice became active 24 and 72 h after the last L-DOPA injection. DD mice exhibited a similar amount of locomotor activity as wildtype mice 24 h after the last L-DOPA injection. Although DD mice presented a decrease in activity 72 h after the last L-DOPA injection, they maintained dark phase-stimulated locomotor activation. Despite low levels of dopamine in DD mice, they exhibited feeding behavior that was similar to wildtype mice. Although grooming and rearing behavior significantly decreased, DD mice retained their ability to perform these activities. Haloperidol treatment significantly suppressed all of these behaviors in wildtype mice but not in DD mice. These results indicate that DD mice maintain some aspects of light/dark phase-dependent spontaneous activity despite dopamine depletion, suggesting that compensatory dopamine-independent mechanisms might play a role in the DD mouse phenotype.

## Introduction

Dopamine is a neurotransmitter that plays important roles in various behaviors, including motor movement, motivation, reward, and cognition [1–3]. Dopamine is also involved in the regulation of circadian rhythm [4]. The functions of dopamine are exerted by its release from dopaminergic neurons in the central nervous system.

The dopaminergic pathway in the basal ganglia is considered essential for motor movement. In Parkinson’s disease patients, a reduction of dopamine concentrations in the striatum that is caused by the degeneration of dopaminergic neurons in the substantia nigra pars compacta leads to motor impairment [5]. The removal of dopaminergic neurons by neurotoxin application leads to motor impairment [6, 7]. The blockade of dopaminergic neurotransmission with dopamine receptor antagonists also results in motor impairment [8]. In contrast, dopamine transporter knockout mice, which have high levels of dopamine, exhibit hyperactivity [9]. Based on these observations, dopamine concentrations may be correlated with the extent of locomotor activity.

The role of dopamine in motor function was investigated using a dopamine-deficient (DD) mouse model [10, 11]. DD mice are genetically manipulated to not produce dopamine. They lack the tyrosine hydroxylase (TH) gene, but TH expression is rescued in noradrenergic neurons to prevent disruptions of norepinephrine and epinephrine. DD mice die within 30 days after birth because of starvation [12]. However, they can live, grow normally, and become adults when they receive daily injections of L-3,4-dihydroxyphenylalanine (L-DOPA), a precursor of dopamine, beginning at 2 weeks of age.

Unexpectedly, we previously found that DD mice became hyperactive in a novel environment 72 h after the last L-DOPA injection, when extracellular dopamine concentrations in the brain were almost completely depleted [13]. In contrast, 24 h after the last L-DOPA injection, when extracellular dopamine concentrations in the brain were low, DD mice did not become hyperactive. These observations indicate that dopamine concentrations are not always correlated with the extent of locomotor activity, and a dopamine-independent motor control system may be involved in hyperactivity in a novel environment.

DD mice did not initially exhibit hyperactivity in their home cage, but they later exhibited hyperactivity ~10 h after the last L-DOPA injection and then became hypoactive thereafter [12]. Our previous study also showed that DD mice were hypoactive in their home cage but not in a novel environment 72 h after the last L-DOPA injection [13]. However, DD mice became active again ~24 h after the last L-DOPA injection [14]. The mechanisms that were associated with the second wave of activity were unclear but might be attributable to the maintenance of light/dark phase-dependent spontaneous activity despite dopamine insufficiency.

In the present study, we focused on the association between the 12 h/12 h light/dark cycle and home cage activity in DD mice. We examined home cage activity in DD mice 24–43 h and 72–91 h after the last L-DOPA injection and examined spontaneous behaviors. Although DD mice were hypoactive during the light phase of the light/dark cycle, they became active when the dark phase began (i.e., the active phase in mice). Dopamine receptor blockade did not significantly inhibit spontaneous behavior in DD mice in the dark phase, suggesting that dopamine-independent mechanisms may play a compensatory role in maintaining circadian rhythm-regulated spontaneous activity that is controlled by the light/dark cycle.

## Methods

### Mice

DD mice were created as described previously [11]. We used wildtype and DD mouse littermates from crosses of heterozygous/heterozygous DD mice on a C57BL/6 J genetic background. The experimental procedures and housing conditions were approved by the Institutional Animal Care and Use Committee (Animal Experimentation Ethics Committee of Tokyo Metropolitan Institute of Medical Science; approval no. 12–43). All of the animals were cared for and treated humanely in accordance with our institutional animal experimentation guidelines. All of the mice were housed in an animal facility that was maintained at 23°C ± 1 °C and 55% ± 5% relative humidity under a 12 h/12 h light/dark cycle (lights on at 8:00 AM, lights off at 8:00 PM). Food and water were available ad libitum. For routine maintenance of the DD mice, 50 mg/kg L-DOPA (Sigma Aldrich, St. Louis, MO, USA) dissolved in 2.5 mg/ml ascorbic acid (Sigma Aldrich, St. Louis, MO, USA) solution in saline was intraperitoneally (i.p.) administered six times per week. DD mice were given paste-type food or DietGel (Clear H2O, Westbrook, ME, USA), in addition to usual food pellets. We examined both male and female 10–30 week old mice.

### Locomotor activity assessment

Locomotor activity was measured with a Supermex apparatus (Muromachi Kikai, Tokyo, Japan) and a sensor monitor that was mounted above the chamber. All locomotor activity counts were automatically summed and recorded every 10 min. DD mice received their last L-DOPA (50 mg/kg) injection at 2:00 PM the day before the test or 3 days before the test. Each mouse was housed in its home cage on the Supermex apparatus 3 h before the locomotor activity test (24 or 72 h after the last L-DOPA injection) to habituate them to the environment. The locomotor activity test was performed from 5:00 PM to 9:00 AM, 27–43 h or 75–91 h after the last L-DOPA injection. To analyze the effect of haloperidol treatment, the mice received their last L-DOPA injection at 2:00 PM the day before the test and were subcutaneously injected with 1 mg/kg haloperidol (Sigma Aldrich, St. Louis, MO, USA) dissolved in 0.08% lactic acid (Wako Pure Chemical Industries, Osaka, Japan) at 5:00 PM (i.e., immediately before the locomotor activity test).

### Spontaneous activity assessment

Spontaneous home cage behavior was recorded with a video camera. During recording, DietGel was used for feeding. For mice that did not receive a haloperidol injection, we evaluated the video that was recorded during the first 6 h of the dark phase (8:00 PM to 2:00 AM). For mice that received a haloperidol injection, we evaluated the video that was recorded during the first 3 h of the dark phase (8:00 PM to 11:00 PM). We analyzed the following spontaneous behaviors: eating, grooming, and rearing. Eating behavior was defined as the mouse placing its mouth in contact with the DietGel. For eating and grooming behavior, the number of bouts and duration were recorded. If the interval between two bouts was >5 s, then they were counted as separate bouts. Rearing behavior was defined as the mouse standing on its hind legs. For rearing behavior, only the number of bouts was counted. Patterns of grooming behavior during the first 2 h of the dark phase were analyzed according to a previous report [15] with some modifications. The number of grooming bouts for each of five anatomical areas (i.e., forepaws, nose/face, head, body, hind legs/tail/genitals) was counted. The percentage of the number of bouts for each anatomical area relative to the total number of grooming bouts was calculated.

### Statistical analysis

The time course data are presented as mean ± SE. The other data are shown as box plots. For multiple comparisons, the data were analyzed using the Kruskal-Wallis test. To perform a comparison of each pair, the data were analyzed using the Steel-Dwass test. For comparisons between two groups, the data were analyzed using the Mann-Whitney two-tailed U-test.Values of *p* < 0.05 were considered statistically significant. The data were analyzed using BellCurve for Excel software (Social Survey Research Information, Tokyo, Japan).

## Results

### Locomotor activity in the home cage increased in both wildtype mice and DD mice during the dark phase

**Fig. 1:**
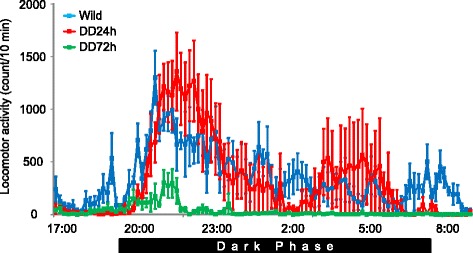
Locomotor activity in the home cage in wildtype mice and DD mice 24 h (DD24h) and 72 h (DD72h) after the last L-DOPA injection (*n* = 6 per group). The dark phase was from 8:00 PM to 8:00 AM. The data are expressed as mean ± SEM

We first examined locomotor activity in the home cage in wildtype mice and DD mice. DD mice were hypoactive during the light phase (5:00 PM to 8:00 PM) 24 and 72 h after the last L-DOPA injection. DD mice 24 h after the last L-DOPA injection became active immediately after beginning the dark phase and remained active during the dark phase (Fig. 1). Activity in DD mice 24 h after the last L-DOPA injection was not significantly different from wildtype mice. The time course of locomotor activity was similar to wildtype mice, indicating that activity in DD mice was associated with the light/dark cycle. DD mice 72 h after the last L-DOPA injection also became active after the beginning of the dark phase while the extent of locomotor activity was less than wildtype and DD mice 24 h after the last L-DOPA injection (Fig. 1), indicating that the dark phase-dependent increase in locomotor activity remained even with extremely low dopamine levels.

### Eating behavior was normal but food intake significantly decreased in DD mice

**Fig. 2:**
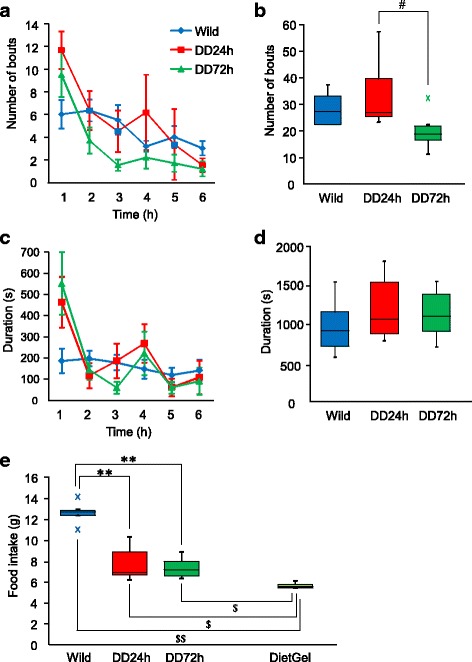
Eating behavior. **a** Time course analysis of frequency of eating during the first 6 h of the dark phase. The data are expressed as mean ± SEM. **b** Box plot showing total number of eating bouts during the first 6 h of the dark phase. ^#^ *p* < 0.05, compared with DD mice 24 h after the last L-DOPA injection. **c** Time course analysis of the duration of eating during the first 6 h of the dark phase. The data are expressed as mean ± SEM. **d** Box plot showing total duration of eating during the first 6 h of the dark phase. **e** Box plot showing food intake and amount of water evaporation from DietGel from 2:00 PM to 9:00 AM. **p < 0.01, compared with wildtype mice; ^$^ *p* < 0.05, ^$$^ *p* < 0.01, compared with DietGel

Activity was higher during the first half of the dark phase in wildtype mice and DD mice 24 h after the last L-DOPA injection. We next investigated spontaneous behavior in DD mice by recording activity in the first 6 h of the dark phase. We first analyzed eating behavior. The number of feeding bouts in the first hour was high and then gradually decreased in wildtype and DD mice (Fig. 2a), although no significant difference was observed between wildtype and DD mice. The total number of feeding bouts in the first 6 h of the dark phase was higher in DD mice 24 h after the last L-DOPA injection compared with DD mice 72 h after the last L-DOPA injection, with no significant difference between wildtype and DD mice (Fig. 2b). The duration of eating behavior was not significantly different between wildtype and DD mice (Fig. 2c, d). These results indicate that DD mice, even 72 h after the last L-DOPA injection, did not lose their motivation to eat. We next investigated whether DD mice actually ate their food. We used DietGel because DD mice have difficulty eating hard food pellets. Approximately 5 g of water evaporated from the DietGel (Fig. 2e). After taking into account water evaporation, the amount of DietGel significantly decreased in DD mice both 24 and 72 h after the last L-DOPA injection (Fig. 2e), indicating that DD mice ate it. However, total food intake was significantly lower in DD mice than in wildtype mice (Fig. 2e). DD mice lost more than 1 g of their body weight if L-DOPA injections were skipped for 1 day. These results indicate that DD mice ate their feed although they exhibited dopamine depletion, but their food intake was less compared with wildtype mice.

### Grooming behavior decreased and patterns of grooming behavior were altered in DD mice

**Fig. 3:**
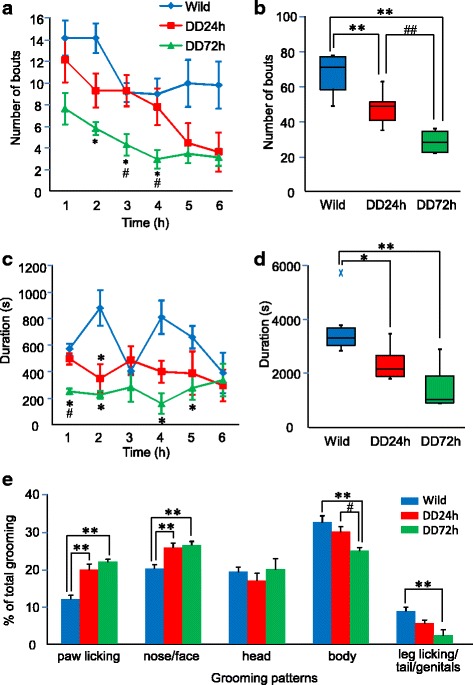
Grooming behavior. **a** Time course of frequency of grooming behavior during the first 6 h of the dark phase. The data are expressed as mean ± SEM. *p < 0.05, compared with wildtype mice; ^#^ *p* < 0.05, compared with DD mice 24 h after the last L-DOPA injection. **b** Box plot showing total number of bouts of grooming during the first 6 h of the dark phase. **p < 0.01, compared with wildtype mice; ^##^ *p* < 0.01, compared with DD mice 24 h after the last L-DOPA injection. **c** Time course of duration of grooming behavior during the first 6 h of the dark phase. The data are expressed as mean ± SEM. *p < 0.05, compared with wildtype mice; ^#^ *p* < 0.05, compared with DD mice 24 h after the last L-DOPA injection. **d** Box plot showing total duration of grooming during the first 6 h of the dark phase. *p < 0.05, **p < 0.01, compared with wildtype mice. **e** Percentage of patterns of grooming behavior relative to total grooming during the first 2 h of the dark phase. The data are expressed as mean ± SEM. **p < 0.01,compared with wildtype mice; ^#^ *p* < 0.05, compared with DD mice 24 h after the last L-DOPA injection

We next investigated grooming behavior. We did not observe a significant difference in the time course of the number of grooming bouts between wildtype mice and DD mice 24 h after the last L-DOPA injection, whereas DD mice 72 h after the last L-DOPA injection exhibited a significant decrease in the number of grooming bouts in the second, third, and fourth hours of the dark phase (Fig. 3a). The total number of grooming bouts in the first 6 h of the dark phase significantly decreased in DD mice. DD mice 72 h after the last L-DOPA injection presented the fewest grooming bouts (Fig. 3b). The duration of grooming behavior was significantly less in DD mice 24 h after the last L-DOPA injection than in wildtype mice in the second hour of the dark phase. The duration of grooming behavior was significantly less in DD mice 72 h after the last L-DOPA injection than in wildtype mice in the first, second, fourth, and fifth hours of the dark phase (Fig. 3c). The total duration of grooming behavior in the first 6 h of the dark phase also significantly decreased in DD mice. DD mice 72 h after the last L-DOPA injection exhibited the shortest duration of grooming (Fig. 3d). Although the amount of grooming behavior may be correlated with dopamine levels, these results indicate that DD mice, even 72 h after the last L-DOPA injection, engaged in grooming behavior despite having extremely low levels of dopamine.

Patterns of grooming behavior are affected by various stimuli, including stress and gene modification [15, 16]. We evaluated patterns of grooming behavior in DD mice. DD mice presented more forepaw and nose/face grooming, accompanied by a decrease in dopamine levels (Fig. 3c), indicating that DD mice spent more time grooming the cranial side of the body than the caudal side.

### Rearing behavior significantly decreased in DD mice

**Fig. 4:**
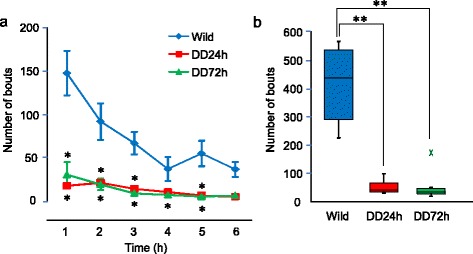
Rearing behavior. **a** Time course of frequency of rearing behavior during the first 6 h of the dark phase. The data are expressed as mean ± SEM. *p < 0.05, compared with wildtype mice. **b** Box plot showing total number of bouts of rearing during the first 6 h of the dark phase. **p < 0.01, compared with wildtype mice

We also investigated the frequency of rearing behavior. Wildtype mice explored the vertical sides of the cage and reared frequently (Fig. 4a, b). DD mice exhibited significantly less rearing behavior 24 and 72 h after the last L-DOPA injection (Fig. 4a, b). Hanging from the wire top of the cage was seen in all wildtype mice, whereas only one of the six DD mice both 24 and 72 h after the last L-DOPA injection hung from the wire top of the cage during the first 6 h of the dark phase.

### Increase in locomotor activity during dark phase was maintained after haloperidol treatment in DD mice

**Fig. 5:**
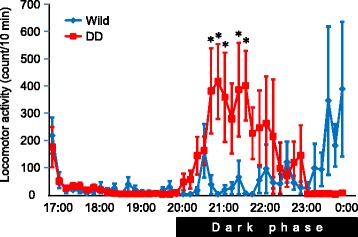
Effect of haloperidol on locomotor activity. Haloperidol was injected at 5:00 PM. The dark phase began at 8:00 PM. The data are expressed as mean ± SEM. *p < 0.05, compared with wildtype mice

To investigate whether the increase in locomotor activity during the dark phase was dopamine-dependent, wildtype mice and DD mice were treated with haloperidol 3 h before the dark phase began. Locomotor activity in wildtype mice was almost completely inhibited for approximately 6 h (~11:00 PM) after haloperidol treatment and then gradually increased, likely because the effect of haloperidol subsided (Fig. 5). In contrast, DD mice exhibited an increase in locomotor activity compared with wildtype mice soon after dark phase began (Fig. 5). Haloperidol treatment decreased locomotor activity compared with no treatment in DD mice (see Fig. 1). After haloperidol treatment, DD mice became mostly inactive after 11:00 PM. These results indicated that increase in locomotor activity during dark phase may be partially dopamine-independent in DD mice.

### Eating behavior was maintained after haloperidol treatment in DD mice

**Fig. 6:**
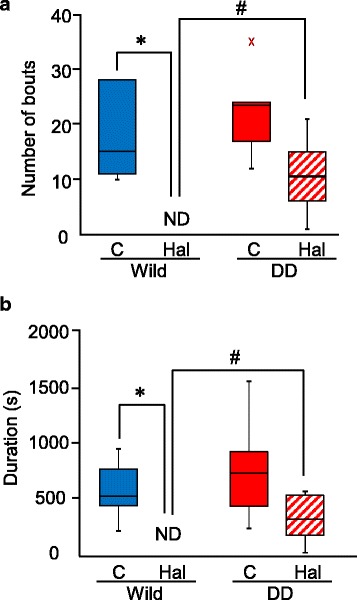
Effect of haloperidol on eating behavior. **a** Box plot showing total number of bouts of eating during the first 3 h of the dark phase. *p < 0.05, compared with control and haloperidol treatment; ^#^ *p* < 0.05, compared with wildtype mice. ND, not determined. **b** Box plot showing total duration of eating during the first 3 h of the dark phase. *p < 0.01, compared with control and haloperidol treatment. ^#^ *p* < 0.05, compared with wildtype mice. C, control; Hal, haloperidol; ND, not determined

The effect of haloperidol lasted until 11:00 PM in wildtype mice. We investigated eating behavior from 8:00 PM to 11:00 PM. Wildtype mice exhibited no eating behavior after haloperidol treatment (Fig. 6a, b). DD mice did not exhibit a significant haloperidol-induced decrease in eating behavior (Fig. 6a, b), indicating that eating behavior in DD mice might be partially regulated by dopamine-independent mechanisms.

### Haloperidol significantly decreased grooming and rearing behavior in wildtype mice but not in DD mice

**Fig. 7:**
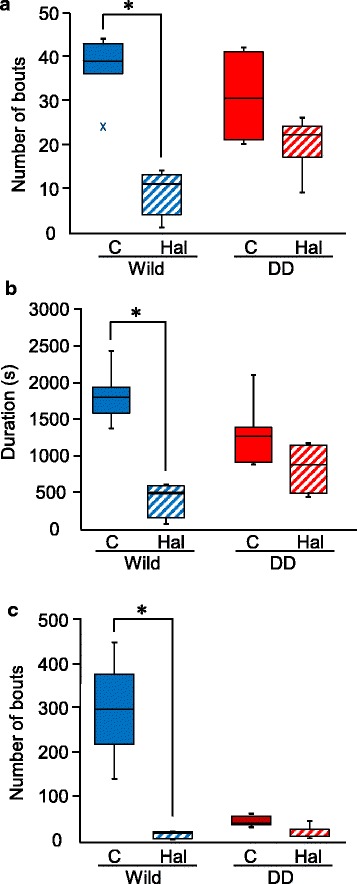
Effect of haloperidol on grooming and rearing behavior. **a** Box plot showing total number of bouts of grooming during the first 3 h of the dark phase. *p < 0.05, compared with control and haloperidol treatment. **b** Box plot showing total duration of grooming during the first 3 h of the dark phase. *p < 0.05, compared with control and haloperidol treatment. **c** Box plot showing total number of bouts of rearing during the first 3 h of the dark phase. *p < 0.05, compared with control and haloperidol treatment. C, control; Hal, haloperidol

Haloperidol treatment significantly decreased the frequency (Fig. 7a) and duration (Fig. 7b) of grooming behavior in wildtype mice. Haloperidol did not significantly decrease grooming behavior in DD mice (Fig. 7a, b). Haloperidol significantly decreased rearing behavior in wildtype mice but not in DD mice (Fig. 7c). Thus, DD mice were less sensitive than wildtype mice to the effects of haloperidol treatment on grooming and rearing behavior.

## Discussion

Spontaneous activity in the home cage in DD mice was almost completely suppressed during the light phase of the light/dark cycle, but DD mice became active when the dark phase began. A previous study found that DD mice became active 24 h after the last L-DOPA injection [14]. In the present study, light/dark phase-dependent spontaneous activity in DD mice was a novel finding. Two possibilities may explain light/dark phase-dependent spontaneous activity in DD mice. One possibility is that a blackout stimulus increases locomotor activity. Another possibility is that circadian rhythm may remain intact in DD mice. Dopamine plays a role in modulating circadian rhythm and is involved in regulating gene expression that is related to circadian rhythm, body temperature, and hormone secretion [4]. Dopamine dysfunction in Parkinson’s disease patients is associated with disturbances in circadian rhythm [17, 18]. In a previous study, nonhuman primates were subjected to 1-methyl-4-phenyl-1,2,3,6-tetrahydopyridine (MPTP)-induced lesions of dopaminergic neurons. The results showed that visual cues of lightness and darkness were required to maintain circadian rhythm, and continuous light exposure led to the disappearance of circadian rhythm [19]. To clarify whether a blackout stimulus is required to increase spontaneous activity or whether normal circadian rhythm is retained, future studies need to investigate spontaneous activity in DD mice under constant dark or light conditions.

DD mice 24 h after the last L-DOPA injection exhibited spontaneous activity that was similar to wildtype mice during the dark phase. Although the amount of some aspects of activity significantly decreased, spontaneous activity did not disappear in DD mice even 72 h after the last L-DOPA injection. A previous study reported that the levels of extracellular dopamine in the striatum in DD mice 24 h after the last L-DOPA injection were less than 2% of wildtype mice [13]. Moreover, extracellular dopamine levels in the striatum were below the limit of detection in DD mice 72 h after the last L-DOPA injection [13]. Such a low level of dopamine would be sufficient to lead to severe motor symptoms of Parkinson’s disease. A previous study showed that 80% dopamine depletion resulted in motor symptoms of Parkinson’s disease [20]. DD mice may have retained spontaneous locomotor activity because compensatory mechanisms may have been engaged. DD mice have intrinsically low dopamine levels beginning at birth, and such long-term dopamine depletion may trigger the activation of compensatory pathways. Constitutive dopamine D_2_ receptor knockout mice did not present hypoactivity, but inducible knockout mice presented hypoactivity, indicating that compensatory mechanisms may be engaged in constitutive D_2_ receptor knockout mice [21]. Moreover, acute depletion of dopaminergic neurons by high dose of 6-hydroxydopamine (6-OHDA) treatment caused severe motor impairment whereas gradual depletion of dopaminergic neurons by low dose of 6-OHDA treatment did not [22]. Compensatory dopamine-independent mechanisms may be responsible for spontaneous activity in DD mice.

Spontaneous activity was not significantly suppressed by haloperidol treatment in DD mice, whereas spontaneous activity was significantly inhibited in wildtype mice. Although haloperidol mainly blocks dopamine D_2_ receptors, spontaneous activity that likely is regulated by dopamine was blocked by haloperidol treatment in wildtype mice. One possibility is that dopamine-dependent activity was blocked by haloperidol treatment. Therefore, spontaneous activity in DD mice might be partially regulated by dopamine-independent mechanisms. Alternatively, low levels of dopamine might alter the function of dopamine receptors or upregulate dopamine receptors, resulting in lower sensitivity to haloperidol treatment.

Dopamine is involved in regulating feeding behavior [23]. Neurotoxin-induced disruptions of dopaminergic neurons resulted in hypophasia [24], and dopamine receptor blockade induced hypophasia [25]. Excessive dopamine concentrations that are induced by psychostimulant treatment reduced food intake [26]. Moreover, dopamine insufficiency was shown to cause obesity [27]. The mechanisms of dopamine’s involvement in feeding behavior have not yet been clearly demonstrated. Previous studies showed that DD mice became hypophasia 10 h after L-DOPA treatment [12, 14, 28], indicating that dopamine is essential to maintain sufficient food intake. Hypophasia in DD mice was rescued by the recovery of TH gene expression in the dorsal striatum [14, 29]. Interestingly, DD mice showed eating behavior when food was served after food deprivation 24 h after the last L-DOPA injection [14]. The experimental protocol in this previous study [14] was different from the present study. Nonetheless, the results of both studies support the hypothesis that the motivation to eat is maintained even with dopamine insufficiency, but overall food intake decreases. Parkinson’s disease patients suffer from dysphagia, and some patients can be treated with dopaminergic stimulation [30, 31]. Moreover, 6-OHDA-treated rats exhibited dysphagia [32]. Dopamine deficiency may decrease the ability to swallow, which would lead to a decrease in food intake in DD mice.

Dopamine is also involved in grooming behavior. The blockade of dopamine transmission by a dopamine receptor antagonist reduced grooming behavior [33]. Both the frequency and duration of grooming behavior significantly decreased in DD mice. The pattern of grooming behavior was also affected in DD mice, in which the proportion of paw licking and nose/face grooming increased while body/leg licking and tail/genital grooming decreased. A previous study reported that dopamine D_1_ agonist treatment increased the grooming of flank regions, whereas D_2_ agonist treatment increased genital grooming compared with control rats [34]. Altogether, these findings suggest that the dopamine might be important for patterns of grooming behavior. Mice that were subjected to the stress of light exposure exhibited an increase in grooming rostral areas [15]. One possibility is that DD mice are more vulnerable and sensitive to external stress than wildtype mice. A significant decrease in rearing behavior was observed in DD mice, which is consistent with previous studies [32, 35]. Dopamine levels may be correlated with the extent of vertical movement [22]. In the present study, the significant decrease in rearing behavior was associated with low extracellular dopamine levels in DD mice.

## Conclusions

In conclusion, DD mice maintained similar light/dark phase-dependent spontaneous activity as wildtype mice, suggesting that compensatory mechanisms may be engaged in DD mice. The decrease in the sensitivity to haloperidol treatment indicates that spontaneous activity in DD mice may be partially controlled by dopamine-independent mechanisms.

## Abbreviations

6-OHDA: 6-hydroxydopamine
DD: dopamine deficient
L-DOPA: L-3,4-dihydroxyphenylalanine
TH: tyrosine hydroxylase
